# P-1630. Filling the Gaps in Sentinel Surveillance: Estimating National and Sub-national COVID-19 Hospitalisation Rates using Wastewater Surveillance in the United States

**DOI:** 10.1093/ofid/ofaf695.1806

**Published:** 2026-01-11

**Authors:** Marco A Gallotta, Julius Arthur Kuebel, Emma Maynard, Markiyan Mitchyn, Richard Bennett, Stefan P Rautenbach

**Affiliations:** Airfinity ltd., London, England, United Kingdom; Airfinity, London, England, United Kingdom; Airfinity, London, England, United Kingdom; Airfinity, London, England, United Kingdom; Airfinity, London, England, United Kingdom; Airfinity, London, England, United Kingdom

## Abstract

**Background:**

The United States of America transitioned to a sentinel surveillance system to track COVID-19 levels in May 2024. National COVID-19 hospitalisation data are now derived from a limited number of sentinel hospitals. Most states no longer report detailed disease metrics, limiting the understanding of localised disease trends across the US. Wastewater surveillance offers a powerful tool to fill these gaps. In states lacking hospitalisation reporting, wastewater data can reflect hospitalisation trends. A strong linear correlation between wastewater levels and hospitalisation rates (Pearson correlation coefficient ranging from 0.60 to 0.94) was observed across the 12 states that report both metrics. This consistent relationship has been the foundation for predictive modelling of hospitalisations in states with only wastewater data.

**Methods:**

A Bayesian hierarchical model was developed to estimate hospitalisation rates from wastewater using state-level COVID-19 hospitalisation data and SARS-CoV-2 wastewater concentration from the CDC between June 2024 and March 2025. The model was fit to z-scored wastewater data and log-transformed hospitalisations using an NUTS MCMC sampler. Posterior samples were used to predict hospitalisations, with results back-transformed to give point estimates and credible intervals.

**Results:**

The model showed high predictive accuracy (modelled hospitalisation 2% lower than observed over the period tested) and captures both the timing and magnitude of COVID-19 burden peaks. Expanding this approach nationwide, the model now provides burden estimates in 29 additional states, significantly improving COVID-19 surveillance.

**Conclusion:**

These state-level estimates enable a more accurate national burden assessment, especially for the latest weeks of available data, where sentinel surveillance estimates may be sparse or delayed. Leveraging wastewater surveillance can offer a robust methodology for real-time estimation of COVID-19 hospitalisations and short-term trends, supporting more effective allocation of healthcare resources and preparedness efforts. This enhances situational awareness and supports data-driven decision-making for public health authorities and treatment providers.

**Disclosures:**

All Authors: No reported disclosures

